# Atorvastatin inhibits cholesterol-induced caspase-3 cleavage through down-regulation of p38 and up-regulation of Bcl-2 in the rat carotid artery

**DOI:** 10.5830/CVJA-2017-005

**Published:** 2017

**Authors:** Roshanak Bayatmakoo, Parichehreh Yaghmaei, Nadereh Rashtchizadeh, Mehdi Farhoudi, Pouran Karimi

**Affiliations:** Department of Biology, Science and Research Branch, Islamic Azad University, Tehran, Iran; Department of Biology, Science and Research Branch, Islamic Azad University, Tehran, Iran; Biotechnology Research Centre, Tabriz University of Medical Sciences, Tabriz, Iran; Neurosciences Research Centre (NSRC), Tabriz University of Medical Sciences, Tabriz, Iran; Neurosciences Research Centre (NSRC), Tabriz University of Medical Sciences, Tabriz, Iran

**Keywords:** atherosclerosis, Bcl-2 protein, cholesterol, caspase-3, p38 mitogen-activated protein kinase

## Abstract

**Aim:**

Atherosclerotic lesions in the carotid arteries lead to a broad range of cerebrovascular disorders such as vascular dementia and ischaemic stroke. Recent studies have verified the beneficial role of atorvastatin (AV) in atherosclerosis. Despite a large body of studies, the mechanisms underlying this effect have not been completely explained. In this study, several experiments were performed on atherosclerotic rat models to investigate the anti-inflammatory and anti-apoptotic effect of AV in the carotid artery.

**Methods:**

In this experimental study, 40 male Wistar rats (250 ± 25 g) were randomly divided into four groups: rats on a normal diet (ND; n = 10); a high-cholesterol diet (HD; n = 10); a high-cholesterol diet plus AV (HD + AV; n = 10); and the AV control group (AV; n = 10). Cleavage of caspase-3 protein, expression of B-cell lymphoma 2 (Bcl-2) as well as phosphorylation of p38 mitogen-activated protein kinase (MAPK) were determined by immunoblotting assay in the carotid artery homogenate. Plasma atherogenic indices, including total cholesterol (TC), high-density lipoprotein cholesterol (HDL-C) and low-density lipoprotein cholesterol (LDL-C) were measured by colorimetric assay at the end of the experiment. Plasma levels of oxidised LDL (oxLDL) were measured by sandwich enzyme-linked immunosorbent assay (ELISA).

**Results:**

After eight weeks of feeding with a high-cholesterol diet, an elevated level of oxLDL was observed in the plasma in the HD group compared with the ND group [214.42 ± 17.46 vs 69.13 ± 9.92 mg/dl (5.55 ± 0.45 vs 1.78 ± 0.26 mmol/l); p < 0.01]. AV administration significantly reduced oxLDL levels in the HD + AV compared to the HD group [126.52 ± 9.46 vs 214.42 ± 17.46 mg/dl (3.28 ± 0.25 vs 5.55 ± 0.45 mmol/l); p < 0.01]. Results also showed that compared with the HC group, the HC + AV group had lower levels of p38 phosphorylation (p < 0.05) and higher levels of Bcl-2 expression (p < 0.05). Lower levels of cleaved caspase-3 were observed in the HC + AV group in comparison with the HC group (p < 0.05).

**Conclusions:**

The resultant data suggest that the anti-apoptotic effect of AV could be partially mediated by the pro-inflammatory protein p38 MAPK and the anti-apoptotic protein Bcl-2 in the rat carotid artery. Atorvastatin can therefore be considered a target drug in the prevention or development of atherosclerotic events.

## Introduction

Atherosclerosis is a chronic inflammatory disease involving multiple pathways. It is characterised by atheromatous plaque consisting of a lipid-core lesion located in the sub-intima of the bifurcation of large and medium-sized arteries, such as the carotid and aorta.^1^,^2^ Accumulation of low-density lipoproteins (LDLs) and their oxidised form (oxLDLs), as major carriers of cholesterol, initiate atherogenic events that are followed by the recruitment of inflammatory blood cells.^1^

The results of in vitro studies have revealed that oxidised LDL causes injury to the endothelial cells (EC),3 the mechanism of which is unknown, resulting in necrosis or apoptosis.^4^ Apoptosis refers to the morphological changes exhibited by ‘actively’ dying cells, including DNA fragmentation, chromatin condensation, membrane blebbing and cell shrinkage,^5^ whereas necrosis is rupture of the plasma membrane and cell lysis following cellular swelling.^4^ The signal transduction leading to apoptosis is characterised by a complex array of biochemical pathways, including inflammation, mitochondrial dysfunction and cell proliferation.^6^

Moreover, triggering of mitogen-activated protein kinase (MAPK), which is a classic inflammatory cascade, is required for oxLDL-attributed induction of apoptosis.^7^ Dysregulation of the MAPK pathway during atherosclerosis leads to modified gene expression, which facilitates disease processes.^3^ Three major members of the MAPK family that are entirely involved in atherogenic events are extracellular signal-regulated kinase (ERK), c-Jun kinase (JNK) and p38 MAPK. Among them, p38, a well-known stress kinase, controls foam cell formation and programmed cell death in macrophages, and facilitates the expression of chemokines and adhesion molecules in the endothelial cells.^3^

Recent studies suggest that JNK induces apoptosis by directly phosphorylating BA, BimEL, and BimL.^8^-^13^ In addition, JNK also phosphorylates and thus inactivates the anti-apoptotic Bcl-xL and Bcl-2.^14^-^16^ In contrast to augmented studies on the regulation of Bcl-2 family members by JNK, there is no proof that p38 regulates apoptosis through direct activation/inactivation of Bcl-2 family proteins.^6^,^17^ Moreover, Bcl-2 protein is a major regulator of the intrinsic apoptosis signalling pathway. In recent years it has been elucidated that it modulates the apoptotic events in vascular cells.^7^,^18^

Activation of caspase-3 plays a unique role in apoptosis and is considered the final step prior to DNA fragmentation. Caspase-3 triggers apoptotic DNA fragmentation by cleaving DFF45 (DNA fragmentation factor 45) or ICAD (inhibitor of caspase-activated DNase), which is changed to active DFF40/ CAD (caspase-activated DNase).

Statins, as classic inhibitors of 3-hydroxy-3-methylglutarylcoenzyme A (HMG-CoA) reductase, have been shown to potentiate decreased plasma levels of cholesterol and the ratio of oxLDL to native LDL, leading to attenuation of the development of atherosclerosis.^19^ Recently, the non-cholesterol-lowering effect of statins, including their effect on platelet adhesion,^20^ cytokine release,^14^ and anti-inflammatory effects21 have been explored.

Atorvastatin (AV) is a lipophilic member of the statin family and is mainly recommended for the treatment of hypercholesterolaemia. It has been shown to have antiinflammatory benefits in the coronary arteries,^3^,^19^,^22^ but the effect of AV on the carotid arteries is seldom investigated. Since inflammation and apoptosis are common events in atherosclerosis, we speculated that AV may attenuate cholesterol-induced injuries in carotid tissue via its influence on inflammation and apoptosis in the carotid arteries. We therefore evaluated the effects of AV on the MAPK signalling pathway and apoptosis in this tissue.

## Methods

In this applied, experimental study, 40 male Wistar rats (250 ± 25 g) were obtained from the breeding colony of the Pasteur Institute, Karaj, Iran. The experiment was carried out in 2015 in the laboratories of the Neurosciences Research Centre (NSRC) located at Tabriz University of Medical Sciences, Tabriz, Iran. The study was approved by the ethics committee of Tabriz University of Medical Sciences (approval number: A125345) and conformed to the Guidelines of the National Institute of Health for the Care and Use of Laboratory Animals (NIH Publications No. 80-23).

The animals were kept under controlled conditions at 22 ± 1°C with a 12-hour light:dark cycle and 50–55% relative humidity. They had free access to standard rodent chow and water, and were housed in individual cages for 96 hours before use.

Atorvastatin (purity ≥ 98%) (Lipitor®; Pfizer Inc, New York, NY, USA), cholesterol (purity > 99.9%), and the protease inhibitor cocktail were purchased from Sigma-Aldrich (St Louis, MO, USA). Rabbit anti-Bcl-2, anti-phospho-p38 (anti- P-p38), anti-p38, HRP-conjugated goat anti-rabbit, anti-cleaved caspase-3 and anti-B-actin polyclonal antibodies were obtained from Santa Cruz Biotechnology (Santa Cruz, CA, USA). All biochemical kits for colorimetric assays of plasma lipid profiles were purchased from Zist Chimi Inc (Tehran, Iran).

We used simple randomisation by coin to divide the rats into four dietary groups: normal diet (ND; n = 10), high-cholesterol diet (HD; n = 10), high-cholesterol diet plus AV (HD + AV; n = 10) and the AV control group (AV; n = 10). HD rats received the normal chow diet plus 2% cholesterol (Sigma-Aldrich, No: C8667) whereas the ND group was fed only the normal chow diet. Rats in the AV and HD + AV groups were given AV (20 mg/kg) dissolved in 2 ml warm water before intra-gastric administration.23 All animals had access to food and water ad libitum daily during the experiment.

After eight weeks of feeding the HD diet with the administration of AV, the rats were intraperitoneally anesthetised using xylazine (4 mg/kg; Sigma-Aldrich) and ketamine hydrochloride (10%, 40 mg/kg; Sigma-Aldrich). Following ligation of the left and right common carotid arteries (CCAs), blood samples were withdrawn directly from the heart of the rats and collected in a serum separator tube. The blood was allowed to coagulate for two hours at room temperature and centrifuged (Beckman model L centrifuge) at 3 000 × g for 20 minutes. The serum was saved for biochemical analyses. The CCAs were removed, put into liquid nitrogen and kept at –70°C for immunoblotting analysis.

Biochemical measurement of plasma levels of triglycerides (TG), total cholesterol (TC) and high-density lipoprotein cholesterol (HDL-C) were determined photometrically in a Vitros 5600 autoanalyser (Ortho-Clinical Diagnostics Inc, USA) in the endpoint manner using Ziest Chimi kits (Tehran, Iran). To calculate LDL-C levels, Friedewald’s formula^24^ was applied as follows: LDL-C (mg/dl) = (TC (mg/dl) – TG (mg/dl)) / (5 - HDL-C(mg/dl)).^25^

Western blotting technique was used to evaluate the expression of bcl-2, cleaved caspase-3 and phospho-p38 proteins, based on the Santa Cruz online protocol. A 10% carotid tissue homogenate in RIPA lysis buffer (Sigma) containing protease inhibitor cocktail (Sigma-Aldrich) was prepared after being centrifuged (SW14R, Froilabo, France) at 4°C and 13 000 × g for 15 minutes.

Protein concentration was measured using the Bradford assay (Bio-Rad Laboratories, CA, USA); 10 μg protein was loaded into each well of 4–10% SDS polyacrylamide gel. Following electrophoresis, proteins were blotted onto the membrane (polyvinylidene fluoride, Bio-Rad) and blocked in 3% bovine serum albumin (BSA) in phosphate-buffered saline (PBS) and 0.1% Tween 20 (PBST). Membranes were blotted overnight at 4°C with the following primary antibodies diluted in PBST containing 0.1% Tween: anti-Bcl-2 (N-19) (1:500; catalogue number sc-492), anti-cleaved caspase-3 p11 (h176)-R (1:500; catalogue number sc-22171-R) or anti-P-p38 antibody (Tyr 182) (1:500; catalogue number sc-101759) and anti-p38 antibody (1:500; catalogue number sc-535). After a rinsing step with PBST, the membrane was incubated with a secondary antibody (HRP-conjugated goat anti-rabbit) (1:5000; catalogue number sc-2004).The membranes were then rinsed in PBST containing 0.05% Tween.

The immune complex was detected with a chemiluminescence method using ECL-plus kits (GE Healthcare, USA). B-actin protein expression was used as the loading control. The intensity of the bands was determined and analysed with the Spectrum multispectral imaging system by the Vision Work LS image acquisition and analysis software (UVP, Germany).

Plasma levels of oxLDL were measured by ELISA using a specific ELISA kit (MBS729489, My BioSource Ltd, USA). The coefficient of variation for the assay was 4.4 to 5.6. The coated monoclonal antibody (mAb) was against a conformational epitope in the apolipoprotein B-100 (apoB-100) moiety of LDL. All procedures were carried out according to the manufacture’s instruction.

Briefly, the mAb-coated wells were loaded with 50 μl standards or samples. In order to determine quantitative amounts of oxLDL in the sample, polyclonal antibody (conjugated to horseradish peroxidase) specific for oxLDL, was added to each well. The wells underwent washing three times with PBS following incubation for one hour at room temperature. TMB substrate solution was then added and allowed to react for 20 minutes. The chromogenic reaction was halted by adding the stop solution. The absorbance of the wells was read using a spectrophotometer (Awareness, USA) at 450 nm. The amount of oxLDL concentration in each sample was determined using a standard curve (x = concentration and y = optical density).

## Statistical analysis

The required sample sizes were obtained based on a previous article so no statistical methods were used to determine sample sizes.^26^ Resultant data are presented as means ± standard deviation (SD). The means of the variables and the differences in mean values between the groups were evaluated using the Mann– Whitney U-test. A p-value of < 0.05 was considered significant.

## Results

**Fig. 1.**
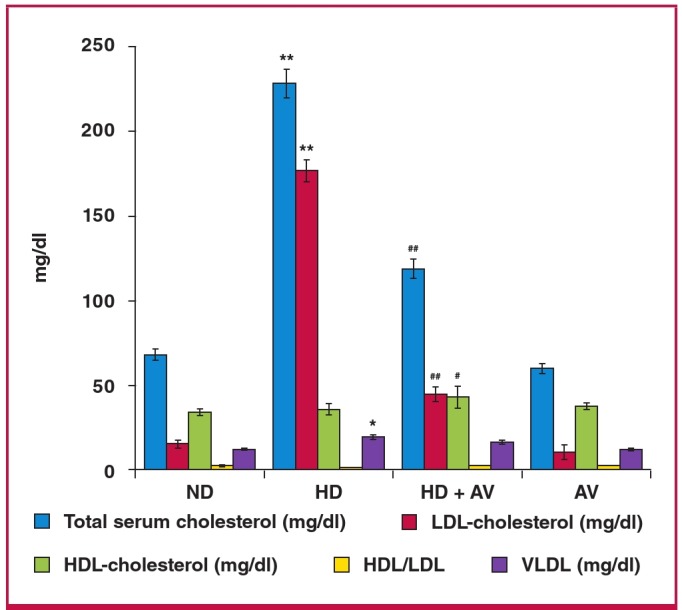
Bar graph showing the attenuating effect of administration of oral atorvastatin (20 mg/kg for eight weeks) on cholesterol-induced serum lipid profiles of triglycerides (TG), total cholesterol (TC), HDL-C and LDL-C in hypercholesterolaemic rats. Data are presented as mean ñ SD; n = 10. *p < 0.05, **p < 0.001 compared with normal diet group; #p < 0.05, ##p < 0.001 compared with hypercholesterolaemic rats using the ANOVA test. ND = normal diet, HD = high-cholesterol (2%) diet, HD + AV = high-cholesterol diet plus AV (20 mg/kg), and AV = AV control group.

We intended to determine perturbations in the plasma lipid profile of the rats in each group to confirm the induction of hypercholesterolaemia after 20 weeks of treatment. As shown in Fig. 1, the serum total cholesterol levels of the HD group were approximately three-fold higher than those of the ND group [229.35 ± 13.26 vs 67.89 ± 5.14 mg/dl (5.94 ± 0.34 vs 1.76 ± 0.13 mmol/l); p < 0.01]. The serum levels of LDL-C in the HD group were more than 10-fold higher than those in the ND group [177.39 ± 10.38 vs 15.20 ± 2.34 mg/dl (4.59 ± 0.27 vs 0.39 ± 0.06 mmol/l); p < 0.05]. A slight but non-significant decrease was also observed in HDL-C levels [31.27 ± 4.69 vs 33.66 ± 2.90 mg/dl (0.81 ± 0.12 vs 0.87 ± 0.08 mmol/l); p = ns].

**Fig. 2.**
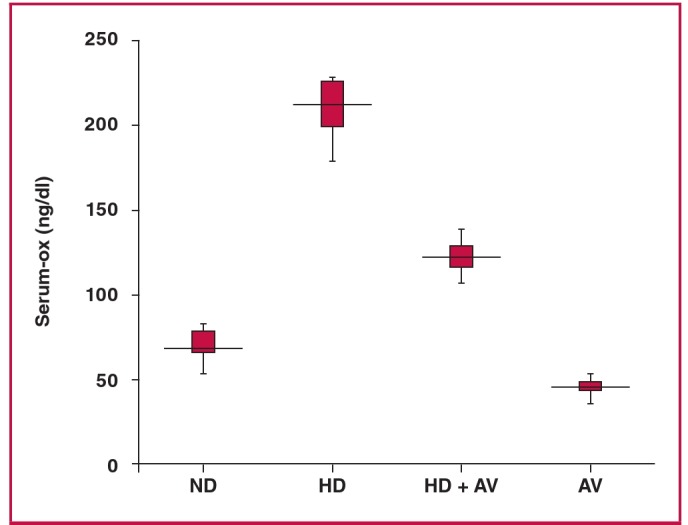
Box plot showing the reducing effect of administration of oral atorvastatin (20 mg/kg for eight weeks) on cholesterol-induced plasma oxLDL levels. Data are presented as mean ñ SD; n = 10. **p < 0.001 compared with normal diet group; ##p < 0.001 compared with hypercholesterolaemic rats using the ANOVA test. ND = normal diet, HD = high-cholesterol (2%) diet, HD + AV = high-cholesterol diet plus AV (20 mg/kg ) and AV = AV control group.

The cholesterol-lowering activity of AV was also evaluated. As shown in Fig. 1, AV displayed a strong cholesterol-lowering activity at a dose of 20 mg/kg. Serum levels of total cholesterol in the HD + AV group decreased approximately 50% in comparison with those of the HD group [119.00 ± 9.187 vs 229.35 ± 13.26 mg/dl (3.08 ± 0.24 vs 5.94 ± 0.34 mmol/l); p < 0.01]. The serum levels of LDL-C in the HD + AV group decreased 75% in comparison to those of the HD group [44.284 ± 6.905 vs 177.398 ± 10.386 mg/dl (1.15 ± 0.18 vs 4.59 ± 0.27 mmol/l); p < 0.01]. The plasma levels of oxLDL were significantly increased in the HD versus the ND group [214.42 ± 17.46 vs 69.13 ± 9.92 mg/dl (5.55 ± 0.45 vs 1.79 ± 0.26 mmol/l); p < 0.01] but AV diminished the plasma levels of oxLDL in the HD + AV group compared to the AV group [126.52 ± 9.46 vs 214.42 ± 17.46 mg/dl (3.28 ± 0.25 vs 5.55 ± 0.45 mmol/l)] (Fig. 2).

**Fig. 3.**
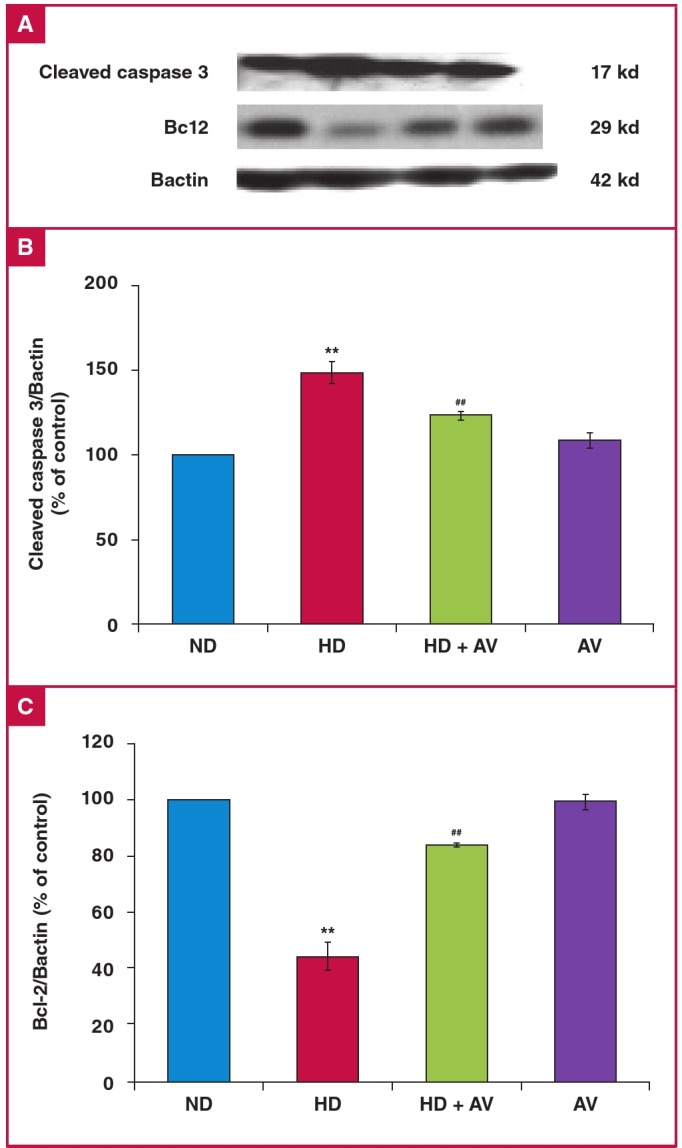
Down-regulation of cleaved caspase-3 and up-regulation of Bcl-2 in the carotid artery of an atherosclerotic rat model after administration of oral atorvastatin (20 mg/kg for eight weeks). A. Immunoblotting of cleaved caspase-3, Bcl-2, and Bactin in ND, HD, HD + AV and AV groups. B. Quantitation of immunoblotting of cleaved caspase-3. C. Quantitation of immunoblotting of Bcl-2. Values are shown as the mean ñ SD of six animals in each group. *p < 0.05.

**Fig. 4.**
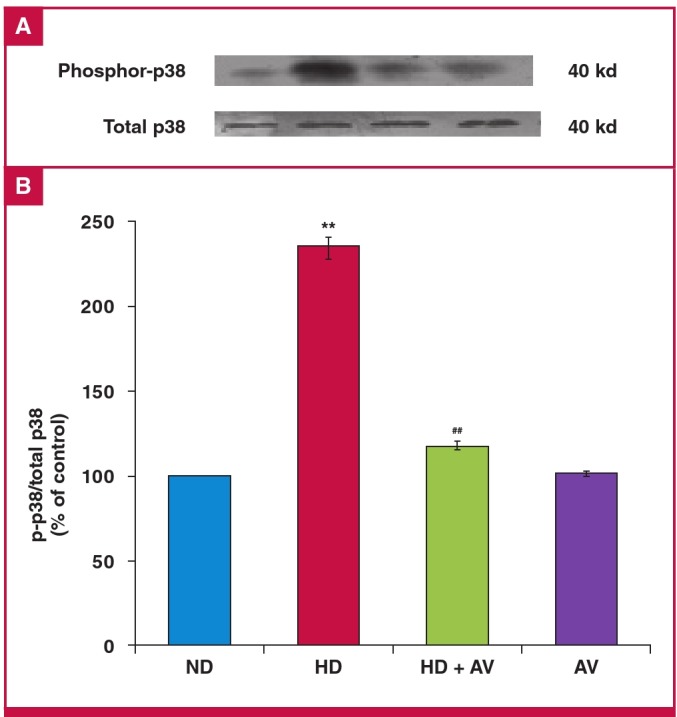
Down-regulation of phosphor-p38 MAPK in the carotid artery of an atherosclerotic rat model after administration of oral atorvastatin (20 mg/kg for eight weeks). A. immunoblotting of phosphor-p38 MAPK, and p38 MAPK in ND, HD, HD + AV and AV groups. B. Quantitation of immunoblotting of phosphor-p38 MAPK. Values are shown as the mean ñ SD of six animals in each group. *p < 0.05.

The protein expression of cleaved caspase-3 and bcl-2, and phosphorylation of p38 were determined to ascertain whether cholesterol induced apoptosis. The protein expression levels of cleaved caspase-3 were measured by Western blotting analysis. Based on the results, cholesterol increased the cleavage of caspase-3 but AV attenuated the cleaved caspase-3 level in the carotid tissuev of hypercholesterolaemic rats (Fig. 3B). Furthermore, to determine whether bcl-2 protein interfered in the cholesterol-induced cleavage of caspase-3, the protein expression levels of bcl-2 were evaluated in all groups. The results showed a significant decrease in bcl-2 level in the HD versus the ND group. Moreover, AV prevented this suppression (Fig. 3C). We then determined the phosphorylation state of p38, a stress kinase, after AV treatment, and found that AV significantly decreased cholesterol-induced phospho-p38 (Fig. 4).

## Discussion

Dysregulation of plasma metabolites and tissue apoptosis are common features of a wide range of degenerative disorders such as atherosclerosis. Risk factors for atherosclerosis, such as oxidative stress, inflammation, hypercholesterolaemia, central obesity and abnormal levels of coagulants often co-exist.

Our experimental study explored the evidence that AV therapy (20 mg/kg), besides its cholesterol-lowering effects, decreased inflammatory and apoptotic events in the carotid artery of an atherosclerotic rat model. We produced the moderate atherosclerotic rat model with the administration of 2% cholesterol [TC = 229.35 ± 13.26 mg/dl (5.94 ± 0.34 mmol/l)], compared with the study by Samout et al. of 1% cholesterol [TC = 90.71 ± 3.08 mg/dl (0.25 ± 0.08 mmol/l)] and that of Beason et al. of 4% cholesterol (TC = 595 ± 429 mg/dl (15.41 ± 11.11 mmol/l).^26^,^27^

We used this model to investigate the expression of inflammation and apoptosis-related proteins in the carotid tissue of hypercholesterolaemic rats after receiving AV. We did not carry out a histopathological study on the carotid tissue, but based on a previous study, even a 1% cholesterol-rich diet is capable of damaging the blood vessels and initiating atherosclerotic events.^28^ Ntchapda et al. showed extensive atherosclerotic plaques were created in almost the whole upper part of the hypercholesterolaemic rat aorta, which was not the case with the normocholesterolaemic rats.^28^

In our study, cholesterol-induced hyperlipidaemia was clearly attenuated after eight weeks of statin treatment. This was because of the cholesterol-lowering effect of AV,^29^ via inhibition of HMG-CoA reductase, the check-point step in cholesterol synthesis.^14^ Moreover, previous studies have shown that up-regulation of LDL receptors on the cell surface is another event that consequently leads to decreased plasma levels of atherogenic LDL particles.^21^

On other hand, based on our findings, cholesterol-induced oxidative stress in the form of plasma oxLDL could be decreased by AV administration, which was confirmed by prior studies.30 Zhang et al. indicated that AV prevented oxLDL-induced oxidative stress in cardiomyocytes via a decrease in plasma levels of oxLDL, inhibition of expression of LOX-1 as oxLDL receptors, and apoptosis.^30^ More recently, Mason et al. showed that in vitro eicosapentaenoic acid (EPA), a triglyceride-lowering agent, inhibited LDL oxidation, and the addition of AV at low concentrations enhanced this inhibition.^31^

Accumulating data from in vitro and in vivo models support the pro-atherogenic role of oxLDLs via: (1) recruitment of polymorphonuclear cells, promoting their transformation into foam cells; (2) induction of the proliferation of smooth muscle cells (SMCs) in the tunica intima; and (3) promotion of apoptosis in the endothelial cells, SMCs and macrophages.^6^ Therefore the oxLDL-lowering potential of AV leads to clinical benefits by attenuating cardiovascular events.^32^

Reduction in oxLDL levels subsequently controls its downstream effectors, such as stress kinases of p38 MAPK and JNK, which consequently reduce scavenger receptors and foam cell formation.33 Confirming previously reported evidence,^14^,^21^,^32^ in our experiments, AV down-regulated cholesterol-induced p38 phosphorylation, which is a pro-inflammatory marker and stress kinase, in the carotid homogenate of hypercholesterolaemic rats. In line with our results, in a concomitant study on the effects of ATO on thrombomodulin (TM), which is critical for vascular thromboresistance, Lin et al. showed in the aorta of cholesterol-fed rabbits, that statins could protect the vasculature from p38-mediated inflammatory damage and that atherosclerosis resulted from cholesterol-dependent or independent mechanisms.^34^

In the study by Rutishauser et al., they showed the beneficial effects of statins on hypertension-induced vascular damage by inhibition of angiotensin II-induced intracellular responses, containing p38 MAPK and RhoA/ROCK activation.^9^ In another study, researchers showed that oxidative stress induced NADPH oxidase production, and p38 MAPK signalling was prevented by statin treatment.^35^ As shown in Fig. 3, cholesterol-induced cleavage of caspase-3 in carotid tissue suggests that activation of the caspase-dependent apoptotic pathway could be negatively influenced by AV, which is similar to the results of our study.

Chen et al., in an experimental rat model of acute myocardial infarction, showed that AV improved left ventricular function and decreased infarct size compared with the control group, along with reduction in the index of cell apoptosis.^36^ Apoptosis is a central component in the pathophysiology of atherosclerosis and is mediated by extrinsic or intrinsic signalling pathways. Bcl-2 proteins act as the major mediators of both apoptosis signalling pathways. Recently it has become clear that they regulate apoptosis in vascular cells following oxidative and inflammatory events, not only by down-regulation of antiapoptotic proteins of Bcl-2 but also by up-regulation of the pro-apoptotic protein of Bax or Bad proteins.^23^

Other findings in our study were that restoration of the anti-apoptotic protein of Bcl-2 occurred after administration of AV. This result is in agreement with previous studies.^8^,^30^ Kutuk et al. considered Bcl-2 protein an important target drug in the treatment of atherosclerosis.^16^ The study by Fröhlich et al. indicated that Bcl-2 had a protective role in fully differentiated ReNcell VM cells.^8^ However, in the study by Peng et al., inhibition of the proliferation of PC3 human prostate cancer cells has been shown via negative regulation of Bcl-2 and positive regulation of p21.^9^

The endoplasmic reticulum (ER), as an important cellular organelle, is implicated in various vital functions of cells,^10^ such as protein folding and translocation,^11^ lipogenesis and control of calcium balance.^12^ The Bcl-2 family, which is found in the ER, controls the many signalling pathways and therefore cell survival.^13^ ER-situated anti-apoptotic proteins such as Bcl-xL and Bcl-2 prevent the effect of a wide range of apoptosis inducers.^18^ Mitochondrial and ER apoptosis signalling pathways can lead to cleavage and activation of caspase-3, a major killer caspase.^18^ The definite output of caspase-3 cleavage is DNA fragmentation, and subsequently programmed cell death.^16^

## Conclusion

The findings of our study, including the beneficial effects of atorvastatin in the suppression of cholesterol-induced cleaved caspase-3 and the concomitant elevation of Bcl-2 and reduction of phosphorylated p38 MAPK, suggest that the anti-apoptotic effect of atorvastatin may be partially mediated by either p38 MAPK or Bcl-2.^18^,^22^ They also suggest that p38 MAPK, a pro-inflammatory protein and Bcl-2, an anti-apoptotic protein, could be targeted in the prevention of cholesterol-induced atherosclerotic events in the carotid tissue by atorvastatin.
